# Organizing a Collaborative Development of Technological Design Requirements Using a Constructive Dialogue on Value Profiles: A Case in Automated Vehicle Development

**DOI:** 10.1007/s11948-017-9877-3

**Published:** 2017-03-08

**Authors:** Steven M. Flipse, Steven Puylaert

**Affiliations:** Science Education and Communication, Faculty of Applied Sciences, Delft University of Technology, Lorentzweg 1, 2628CJ Delft, The Netherlands

**Keywords:** Responsible innovation, Constructive dialogue, Automated vehicles, Co-creation, Design requirements

## Abstract

Following societal and policy pressures for responsible innovation, innovators are more and more expected to consider the broader socio-ethical context of their work, and more importantly, to integrate such considerations into their daily practices. This may require the involvement of ‘outsiders’ in innovation trajectories, including e.g. societal and governmental actors. However, methods on how to functionally organize such integration in light of responsible innovation have only recently started to emerge. We present an approach to do just that, in which we first develop value profiles of the involved actors, and second, design a workshop setting that allows innovators to develop design requirements in collaboration with representatives of parties that are not usually involved in such innovation design practices. Using a case study in automated vehicle development, we positively demonstrate the possibility and utility of our approach. We stress that in this study we wish to demonstrate the functionality of our developed method, and did not search for scientifically valid outcomes regarding this technical field.

## Introduction

Current societal and political pressures for responsible innovation (RI) encourage innovators to adopt more socially responsible innovation practices. However, what that translates to in practical, daily science and engineering work remains somewhat ambiguous. During the past decades, methods and tools have been developed to help innovators become more aware of societal and political concerns, such as constructive technology assessment (Schot and Rip 1997), and methods to feed back such possible concerns directly into on-going innovation development, such as real-time technology assessment (Guston and Sarewitz 2001), value sensitive design (Friedman et al. 2002) and midstream modulation (Fisher and Mahajan 2006). Some scholars have called for ‘constructive dialogue’ between innovators and societal interest groups and possibly otherwise interested or relevant stakeholders (Jokinen 1995; Litz 2008; Nanopodium 2015), so that the values and opinions of those stakeholders can be considered by innovators. Such consideration is needed for innovators in order to answer RI calls, and, following the Stilgoe et al. (2013) model for RI, specifically for addressing the described notions of *inclusion* of stakeholders and their viewpoints, and being *responsive* to their thoughts and ideas by actively incorporating them in innovation development.

However, the term ‘constructive dialogue’ has not really been defined in the literature. Also, methods to identify and distill values from interest groups have not been standardized. In addition, methods for collaborative innovation, actively including external societal actors, have only recently started to be contemplated. In an attempt to address these three issues simultaneously, we present a method that aims to help innovators to identify important societal values, and involve external stakeholders in their innovation process, in order to come to a co-creation process in which the identified viewpoints of the actors can be translated into practical design requirements.

We do so using a case study from the field of automated vehiclesdevelopment, as an exemplary area of science and engineering that is currently active. We do not claim scientific validity and reliability of our results in this particular innovation area, and want to emphasize that we wish to present the *methodology* for setting up a constructive dialogue on value deliberations, as well as what this can result in in innovation practice.

This paper is structured as follows. Section “Background” section describes the theoretical background against which we position our method, starting from the role of values in innovation, to come to a description of criteria for constructive dialogue about values. Section “A case study on automated vehicles “section introduces the case study of automated vehicles, as the subject of our study. Section “Methodology” section introduces the method we developed for identifying and discussing values based on value profiles. Section “Results” section presents the results of our study, not to present scientifically valid and reliable outcomes, but to demonstrate a format for reporting that could be used in other studies. We end this paper with a discussion and conclusion in Sect.”Discussion & conclusion” section.

## Background

### From Values to Design Requirements

Values lead to guidance for our behaviour and help people to make choices (Rokeach 1973). Of course values are culture dependent (Hofstede 2001), change per context (Witesman and Walters 2014), but can be more or less stable (for both an organization and a person) and only change little over time (Abramson and Inglehar 1995). Technological designs embody values (Winner 1980; Flanagan et al. 2008; van de Poel and Kroes 2014), and such designs could strengthen (but also violate) certain values held by other actors (Roeser 2012). E.g., in the transport domain, intelligent speed assist sets a maximum to the speed, strengthening the value of safety, but limiting the autonomy of the driver.

For their implementation and use, technologies have both functional and moral boundary conditions (Flanagan et al. 2008; Van den Hoven et al. 2012). Values of the different actors can lead to input for these boundary conditions. Van de Poel (2013) describes how values can be specified to norms, and norms to design requirements. For example: the value ‘sustainability’ can be specified to the norm ‘less fuel consumption’ and the design requirement ‘an engine of type X’.

But people generally find it hard to discuss values at an abstract level (Oppenhuisen and Sikkel 2000). Translating values into norms and design requirements helps to make these values more tangible in real life (*cf.* the value hierarchy by Van de Poel 2013). Investigating real-life-relevant, context-specific values possibly should be done with a high diversity of actors (Cuppen 2012), as diverse values can be held and set by all these different actors. The following transformation of values into norms is mostly done by designers and/or engineers themselves, but RI asks that this is done in collaboration (Stilgoe et al. 2013), and in particular between experts and other, non-technical experts (Collins and Evans 2002; Van de Poel 2013), since both domain specific knowledge (e.g. information about automated vehicles) and context specific knowledge (e.g. what future drivers want) may be required. In this way morality can be ‘materialised’ (Verbeek 2006), not only by engineers, but by the all relevant actors collaboratively.

But still, designers will probably demand some degrees of design freedom, and may want to be in charge of the transformation from norms to design requirements. This might clash with views on design that encourage involvement of external actors, e.g. using Midstream Modulation (Fisher and Mahajan 2006) or more downstream methods of public engagement to include values of other actors potentially affected by the innovation under development, even though such involvement is potentially valuable.

To overcome this possible misalignment between freedom and RI, approaches like Value Sensitive Design (Friedman and Kahn 2002), the related Values at Play (Flanagan et al. 2008) and Socio-Technical Integration Research (Fisher and Schuurbiers 2013) may provide valuable insights into how an innovation design process can be based upon a more complete set of values. However, in most VSD (Value Sensitive Design) cases in practice, only one actor or one actor other than the designers themselves are used to give input to the designs, usually the prospective user. In the automated vehicle innovation area, but also in many other fields, multiple actors (drivers, but also other road users, infrastructural bodies, legislators that approve or prohibit circumstances of automated vehicle use, road drivers associations, etc.) are influenced by an innovation and possibly they should also be involved in the design process. Still, while VSD studies have mostly been carried out within the software engineering domain (Friedman and Kahn 2002; Friedman et al. 2002; Flanagan et al. 2008; Meijdam 2015), there are also experiences in e.g. robotics (Cheon and Su 2016), wind turbines and wind parks (Oosterlaken 2015), demonstrating its potential value as an approach in both technological artefact development and sociotechnical system development.

As such, VSD principles may be useful starting points for setting up a constructive dialogue about values. We did expand our search for a suitable constructive dialogue method beyond VSD. E.g., within STIR (Socio-Technical Integration Research) and its Midstream Modulation approach, innovators interact with a researcher from the social sciences/humanities (embedded humanist), who helps innovators to break up and analyse (modulate) their innovation-relevant decisions at the laboratory floor, in order to expand their considerations beyond technical aspects. By structuring the collaboration by means of a protocol, the embedded humanists facilitate broader integration of social and societal aspects into innovation practice. Since STIR has mainly been tested within the field of biotechnology and nanotechnology, its transferability to automated vehicles context should be assessed in this study also. Still, we feel that the form of interactional expertise (in contrast to contributory expertise, see Collins and Evans 2002) within STIR could help innovators in their consideration of viewpoints by different actors. In particular the form of Socratic questioning that an embedded humanist should deploy, would be a valuable feature for a person facilitating our constructive dialogue workshop.

Building on the principles of value elicitation in VSD, the interactional expertise of a workshop facilitator in STIR, and the notion of contributory expertise or other actors (*ibid.*) for RI, we developed our value profile discussions in the form of a constructive dialogue workshop. That is what we will present in Sect. 3, after we describe the notion of ‘constructive dialogue.’ The extent to which this combination of methods is appropriate for our context, we discuss later in Sect. 6.

### Criteria for Constructive Dialogue

While the term *constructive dialogue* is mentioned a few times in the literature (see e.g. Jokinen 1995; Litz 2008; Nanopodium 2015), the term itself is not very clearly defined. Therefore, we tried to develop our own criteria. Of course, by definition, methods of constructive dialogue should be ‘constructive’, and be ‘dialogue’. But also, the method should meet innovation specific boundary conditions. These elements are addressed below.

The ‘constructive’ element may have a relation to use of the term in Constructive Technology Assessment (CTA). Traditionally TA (Technology Assessment) investigated the possible consequences new technologies may have (Schot and Rip 1997). This was mostly done by experts, sometimes in cooperation with societal actors. CTA explicitly had not only the goal to assess, but also to influence the final design, similar to what Stilgoe et al. (2013) or Von Schomberg (2013) have in mind with the notion of Responsible Research and Innovation.

But unfortunately CTA seemed to have remained an activity ‘outside the lab’ (Berloznik and Van Langenhove 1998). Later, Real-Time TA was developed which included a broader range of viewpoints, which could also include e.g. opinion polling, aiming to *“elicit values and explore alternative potential outcomes”* and make innovations more *“amenable to understanding and, if necessary, to modification”* (Guston and Sarewitz 2001: 98). This may have helped paving the road towards more collaborative methods, where reciprocal exchange of expertise between stakeholders is the norm (Flipse et al. 2013). These methods include e.g. Ethical Parallel Research (Van der Burg 2009), Sensitisation (Wilsdon et al. 2005; Penders et al. 2009) and Midstream Modulation (Fisher and Mahajan 2006).

While these methods provide ways for innovators to ‘construct’ innovations collaboratively with other actors, allowing also viewpoints of others to be included in the design process, it may not always encompass dialogue directly with the relevant stakeholders. E.g. in Midstream Modulation an embedded humanist collaborates with an innovator, but *direct* input from other societal stakeholders is not included in the method. Still, a person like the embedded humanist could possibly help structure conversations, similar to what is the case in Midstream Modulation, if such conversations would also include other relevant stakehodlers. To be more complete in terms of value considerations, direct and facilitated dialogue with the affected stakeholders, to make their values more explicit, may be recommendable for RI in practice.

The ‘dialogue’ component may simply be translated as the flow of meaning through a group. Davies et al. (2009) distinguish two types, i.e. policy informing dialogue events and dialogue events that do not seek to inform public policy. To influence innovation practice, probably the type we envision has most in common with policy informing dialogue, and embedding dialogue in formal decisions making is important for it to have an effect (*cf.* Joss 1998). Yet of course, there are requirements for such dialogue. We follow Smaling (2008), who explains that a dialogue should meet five criteria: equality, mutual trust and respect, openness, argumentative quality and a reflective nature. In his work he also defines a critical dialogue, which should focus on reflection and cooperation of the participants.

Verhoeff and Kupper (2014) followed up on these notions. Regarding equality, every participant should be able to set the topic and ask questions. For mutual trust and respect, the behaviour and language during the interaction should demonstrate respect to one another, also in being different. The other party should also be honest. Regarding openness, all parties should share relevant knowledge *and* emotions, and try to interpret each other as well as possible. They help each other in formulating arguments. Concerning the argumentative quality, statements from the participants should be based upon acceptable arguments; discussion tricks or fallacies are not used. Regarding reflection, the participants think about their role and see the dialogue as an outcome of the four aforementioned aspects.

Regarding the further innovation specific boundary conditions, first, it should be made explicit that innovation does not occur in isolation, but within an actor network. This requires that the collaborative process during interaction between the stakeholders is multidisciplinary, with the possibility of no common language being present initially. This may initially hinder the dialogue between the actors. Similarly, following the Collingridge (1980) dilemma, there is no explicit and clear definition of the innovation under development, and hence no clear image of the innovation. E.g. in the field of automated vehicles, different actors can have different ideas on what the specifications of a self-driving car may be. Therefore, such common language needs to be developed, while all involved stakeholders acknowledge that there are different ways of ‘knowing’.

Second, as highlighted above, the dialogue method should still offer some degree of freedom for innovators to design the products and services they want; constructive dialogue should not merely be a way to allow for other actors to prohibit, restrict or hijack the innovation process, but to critically inform it.

## A Case Study on Automated Vehicles

### Case Study Rationale

We selected the case study in the field of automated vehicles for various reasons. Apart from one of the authors being a grad student in an automated vehicles research department within our university, our aim was to setup a method for collaborative innovation development. Therefore, we looked for an innovation that people would be able to relate to easily. So no conceptual or enabling technologies such as nanotechnology or biotechnology was regarded as acceptable, but something concrete, with a noticeable, direct effect on society was chosen. Also, the innovation should not be implemented yet, so still be in the design stage, as to make sure that possible stakeholder input can still be taken into consideration in the development of the innovation. We consider the development of automated vehicles to meet these requirements.

### ‘The’ Automated Vehicle

This section further illustrates the dynamics of that case study. Automated driving comes in different types of vehicles and levels of development (SAE International 2014). Early levels 1 and 2 include cars with adaptive cruise control and lane keeping assistance, which are already available, whereas level 5 includes cars without a steering wheel. In between are levels 3 (the driver is able to e.g. read a paper, but needs to take over the steering wheel when asked), and 4 (car can get itself into safety if it isn’t able to handle a difficult driving situation, but there is still a steering wheel).

Literature indicates two likely development paths for the future: *autonomous driving* and *cooperative driving* (Timmer and Kool 2014; Wilmink et al. 2014; Bhat 2014). In the autonomous driving development path, the car drives on its own, and only monitors the world outside (e.g. the Google car, Google 2015). In the *cooperative* path cars monitor the driving environment and communicate with other vehicles and infrastructure. These cooperative cars can drive in groups or trains (e.g. the DAVI or the SARTRE-project, see Volvo Trucks 2012; Hoogendoorn et al. 2014). The former path argues for making the cars drive on their own first, before possible collectivity adjustments are made, whereas the cooperative path argues for directly making the vehicles communicate with each other while they are becoming more and more automated. Probably, not one or the other development path will be chosen ultimately, but the point is that current uncertainty exists about which development path should be chosen, and hence, what the implications will be for automated driving. This uncertainty makes it difficult for policy makers to develop policies, but also for public actors to form an idea of what automated vehicles can be, and what kind of implications they could have on the long run. Considering that the actual form of automated vehicles is not clear, means that input from potential users and other affected stakeholders could still be valuable. And actors from this technological field argue for stakeholder involvement. E.g., Timmer and Kool (2014) argue that the public and interest groups should be part of the discussion as their input can be essential for ‘societal embedding.’

### The Socio-Economic and Socio-Ethical Context of Automated Vehicles

To prepare the respondents and participants who contributed to the empirical part of our study with some background information on the socio-economic and socio-ethical context and related difficulties, we first investigated this context ourselves. Based on the overview presented below, which we shortened considerably for the purpose of this article, the introductory materials that we present later (Sect. 4) were developed.

Frequently car manufacturers, futurists and government representatives paint pictures of a world with fewer accidents, less congestion and lower emissions (O’Brien 2014; Schultz van Haegen 2015; Mercedes- Benz 2015). However, many of these advantages depend on how the car will be programmed: less congestion only arises when shorter intervehicle distances can be held, leading to potentially more dangerous driving situations. And why would there be less emissions if the car becomes a more attractive mode of transport?

Possible societal progress can be split up into personal benefits for the driver and benefits for society as a whole (Anderson et al. 2014). The consumers of a self-driving vehicle may mainly be interested in the personal benefits, but their use of an automated vehicle will also influence other road users. Also, there is no scientific proof (yet) for more efficient traffic flow or improved road safety. And, there are concerns, e.g. *“liability details remain undefined, security concerns linger, and without new privacy standards, a default lack of privacy for personal travel may become the norm”* (Fagnant and Kockelman 2015: 1).

To be clear, we do not argue against the introduction of automated vehicles, but just want to indicate that there is still a large bandwidth in which automated vehicles can be developed that is desirable, and to indicate that developmental uncertainties make it difficult for actors to have an opinion about this technology, and to develop policies on it. When it comes to involvement of stakeholders in a constructive dialogue about its future development and implementation, there are at least four groups of people. These include the manufacturers and users, but also the ‘non-consumers’ (other road user such as the cyclist or the ‘normal car’ owner) and the governmental bodies that regulate admission and facilitate infrastructure.

Of course, there are also insurance companies, public transport companies, research institutes, road user representative organizations, environmental groups, and potentially many parties who could be considered, but for this research we limited ourselves to these four actors, even though the approach we describe below could also be applied to include more stakeholders.

## Methodology

This chapter describe our method along with the rationale for using the steps in this method. In two steps, we present how we came to the constructive dialogue approach based on value profiles, that is meant to critically discuss ways forward and develop design requirements for the car manufacturers. First, we describe how we developed a value profile for each of the actors involved in the dialogue later. Second, we describe how we organised the dialogue to specify values into norms and design requirements. After these two steps, we describe how we analysed if this method meets the requirements for a constructive dialogue we described above. The three consecutive sub-steps for the value profile development and the two consecutive sub-steps for the norm specifications in the dialogue consists of two steps are described in Table 1.

**Table 1:** Methodological overview with research steps, with indications of who was involved

Steps (stakeholders involved)	Goal
1. Value profile per actor	Investigate what actors find important and find value conflicts
1a. Interviews (stakeholder representatives and experts)	Create a list of moral boundary conditions of different actors
1b. Clustering (experts)	Clustering the statements to values
1c. Scoring (respondents from stakeholder groups)	Gain insight in the relative importance of the values per actor
2. Specification from values to norms	Having a constructive dialogue by specifying the values to norms
2a. Scoring together (students who role-play)	Warming-up and creating a common value profile
2b. Specification to norms (idem.)	Specifying the values to norms and design requirements in a constructive dialogue

### 1a. Interviews

As a result of there being no ‘kollektive Vorstellungsbasis’ (Fraedrich and Lenz 2014) around the automated vehicle, people probably all picture something different in their heads when asked what they think of an automated vehicle. This makes survey results on automated vehicles questionable. Many surveys ask questions like: ‘*do you like automated vehicles?*’ or ‘*would you buy an automated vehicle?’* (Casley et al. 2013; Schoettle and Sivak 2014; Kyriakidis et al. 2015; To Connect 2015). But while most of these do explain what type of automated vehicle they mean, many aspects remain unclear regarding e.g. the real effect on traffic flow, business models, and privacy issues.

We therefore argue it is better to formulate survey questions that are more utopian: ‘*what would an ideal automated vehicle look like?’* The differences in image then become clear and concerns and expected benefits can be identified.

That is the first research step, but in a special way. In total 6 interviews were done with real actors. One with representatives of each of the actors (the government, the manufacturers, a likely consumers and a likely non-consumer), and two extra interviews are done with two experts, i.e. members of the Intelligent Transportations System team of the Dutch road driver association, ANWB, who have much knowledge on self-driving cars. As sample saturation frequently occurs after 5 interviews, six interviewees should be enough to get to a relatively complete set of values. In a one-on-one interview, we started by asking what the representatives and experts think about self-driving cars, what they are enthusiastic about, and scared of. Next, some background information of *level 3* automated vehicle is presented to the representatives, as the main topic of our investigation, in part based on the overview provided in Sect.”The socio-economic and socio-ethical context of automated vehicles“ section. After this, they are asked to design a small brochure of their ideal automated vehicle. During the drawing, the representative explains his/her flyer to the interviewer.

To help the representatives in the creative part, they get an A3 paper, as a basis of their brochure. On a separate A4 some starting points are given, including some pictures of cars and some starting sentences like, “We promise”, “This vehicle can”, “Especially for you” or “This car is not”. With scissors and glue they can start designing.

The interview setup was tested three times with students, before we asked the real automated vehicle actor representatives. Once only with questions, once only with the creative part and once with a mix. The mix we described above gave the best results. The interviews were recorded with a smartphone, transcribed, and open coded using QSR NVIVO software. The outcomes of this analysis was a mix of statements on value descriptions, norms, and design requirements, which needed to be clustered into values for our further analyses.

### 1b. Clustering

We developed the clusters of statements into values that are as Mutually Exclusive, Collectively Exhaustive (ME CE, Rasiel 1999) as possible. The ME-part was done in the clustering session with three automated vehicle experts, the CE was done based on a cross-check with existing literature on values regarding automated vehicles, and the insights of another independent expert.

From the codes resulting from the open coding analysis, our three experts created value hierarchies (*cf.* Van de Poel 2013). These experts included two mobility experts from a Dutch national research organization, and one social scientist, all having affinity with values and automated vehicles. The clustering step is precarious as it uses both the codes developed by the researchers and common knowledge of the experts to fill in the gaps in the hierarchy. Such common knowledge is needed, as sometimes the overlapping concepts are named differently in the interviews, and sometimes statements are neither values, norms, or design requirements, and therefore need to be discarded. This approach is systematic, explicit and transparent, yet also hard to reproduce with different actors and not completely irrefutable (*ibid.*). Through inclusion of multiple actors from multiple expertises, we did consider this method to be the most reliable.

The clusters should represent all aspects of automated vehicle as appropriately as possible. Also to make the clusters collectively exhaustive, two checks are done. The first one is a literature review on values to check if all values fit in the clusters that are made. The second test is another interview, with another independent automated vehicles expert, to confirm that all the statements fit the clusters. After these two checks, the clusters are given descriptions by the researchers.

### 1c. Scoring

The literature covers two general methods to create an order in values: ranking and rating (see also Maio et al. 1996). For small sets of values the rating (giving points) may be preferred (*cf.* Alwin and Krosnick 1985). As we will show later, our analysis resulted in only eight value clusters appearing in the previous step, so we considered rating to be the most suitable method.

**Fig. 1:**
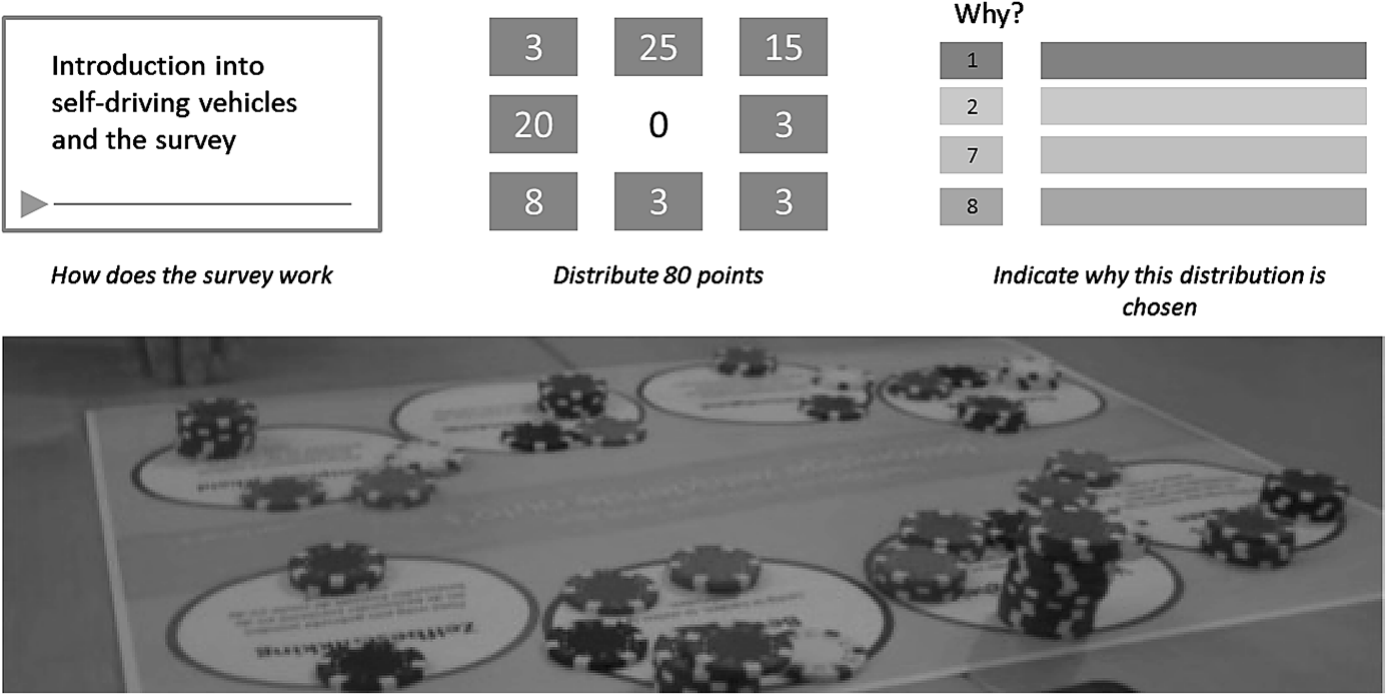
*Top* three consecutive screens used for online data collection, including an introduction video, the constant-sum solving screen, and a form to highlight motivations of participants. *Bottom* the analog system of point distribution using a board with descriptions

We followed the ECHO-framework approach (Michalopoulos et al. 2013) for a multi-criteria assessment using gradual labelling, only in our case the weights of the several aspects are set by the actors in the form of a survey. The participants in the different actor groups are asked to distribute 80 points over the 8 values (10 per identified value). This is essentially a constant sum problem (Survey Analysis 2015), in which a dilemma is created: giving points to one aspect will automatically lead to fewer points being left for others. We used an online survey system that we developed ourselves, as well as a non-digital system with poker chips (see Fig. 1).

The digital links to the online questionnaires are distributed via direct e-mails, social media and relevant newsletter mailing lists. Considering that the automated vehicle is not on the market yet, we provided different instructions for consumer and non-consumer respondents, clearly indicating that they should fill in the questionnaire from that perspective. Since it’s hard to reach government representatives and manufacturers via newsletters, these were invited via direct e-mails to people within the networks of ourselves and our research groups.

The online system consists of five screens, that appear to the invited respondents consecutively. The landing page explains the set-up of the survey, the age of the participant is asked and the role of the respondent is explained (consumer, other road user, government or manufacturer). On the next page an explanation of self-driving cars is given in a movie^1^ and in text.

Next, a matrix shows the 8 values; the middle square counts the points left to divide over the values. For each value a number of points can be filled in. For each value a short explanation is given by hovering; if the respondent needs more information, the ‘info’ button shows a pop-up with a further explanation of that value. The pop-up also briefly explains what it implies when many or few points are given, to further help the respondents.

Hereafter, to see the motivations of the respondents and to check if the survey is understood correctly, the respondents are asked to underpin why they have chosen the two highest scored values and the two lowest scored values. The four required fields on this page are not mandatory to fill out so as to decrease the dropout during the questionnaire. In the final screen, respondents are thanked, asked whether they wish to participate in follow-up research and if they have any other comments.

In the offline version, the researchers (authors of this paper) give the introduction, and the scoring step is done on a scoreboard with 80 poker chips. After the respondents have divided the poker chips, a picture was taken; later the outcomes were administered the same way as the online surveys, but in the offline version no qualitative data is collected on motivations for their highest and lowest scores. This offline version is suitable for places where many potential respondents come together that may otherwise be hard to reach. In our case, we visited the *Back to the future festival* of Connekt, at the 21th of October in Delft, the Netherlands. At this event another 25 participants (mostly Government officials and car and car technology manufacturers) completed the survey.

The outcomes of the datasets were analysed: (1) the value profile per actor group and the differences between the rankings between groups; (2) the score distributions within in the group, looking at standard deviations; (3) a qualitative analysis on the respondents’ motivations for their answers, to check for different interpretations of the values and if they understood the survey questions.

### 2a. Scoring Together

After we generated the actor profiles, the first step of the actual dialogue workshop could take place. This workshop was held three times, one pilot and two sessions. Both sessions were organised with engineering students and graduates who play the roles of the four actors, considering that we could not find real group representatives willing and able to join in.^2^ First, the same information about self-driving cars is given to the participants as for the survey. The participants then divide points over the 8 values, as was done in the offline questionnaire, but now together, using our off-line version with poker chips.

We figured methods like one chip at a time per actor could bring in strategic elements, making the atmosphere less constructive. Therefore, we introduced a two-step setup, in which every participant is given 10 chips to divide themselves all at once over one single board, after which participants are asked to underpin why they divide the points as they did. Hereafter, the group gets another 40 chips to divide together on the same board. The result is the common value profile.

This profile can be compared to the survey results of the different participants. Of course, a possible outcome of the workshops is that the four actors cannot come to consensus on how to do this together, but this is a risk we had to take. We photographed how the chips were divided. The later two sessions were recorded and transcribed, to analyse the discourse later.

### 2b. Specification to Norms

According to Van de Poel (2013: 265) specifying *“helps to trace more precisely the value judgments and possible disagreements”*. This step is where the constructive dialogue should take place and where this statement can be tested. It has some similarities to the Delphi method, in which representatives of the groups can try to create a common value set. But instead of an anonymous, online Delphi method (Linstone and Turoff 2002), we wanted an open discussion on the relative importance of these values. So discussions and motivations given regarding certain point distributions were discussed openly among the participants, in a workshop setting supervised and structured by the researchers.

The outcomes of the value profiles are not the main interest of this research, but rather the process of specification of norms and design requirements. Collectively developing a value hierarchy may help pinpointing exactly where there is disagreement about the specification of values in design. Whereas the value profiles from the questionnaire gave a global view on where the disagreements could be, the specifications should give more in-depth insights. By involving multiple actor perspectives, we hoped to trace discussions on values even better than in previous approaches, even with limited numbers of actor representatives.

During this session, the participants were given an A0-sized paper sheet, markers and instructions on how specifying to norms could work, to further explain what they were required to do. First the value with the most points was specified into design requirements. Next, values which in the first round caused most discussion were specified. The facilitator and the participants together choose which values these were.

In addition to the earlier recordings and photographed profiles, also these sessions were recorded, and the developed A0 posters with specifications were photographed. The discourse was closed coded using QSR NVIVO, to check for indications of constructive dialogue as described earlier (Sect. 2).

## Results

### 1a. Interviews

After transcription and open coding of the interviews we removed duplicate ideas of the actor representatives and experts, resulting in a total of 72 product specifications. We found that many representatives named the same or similar aspects, and that when we asked which of the aspects were more important than others, people found it difficult to answer. Our two ANWB automated vehicle experts were able to identify and describe more aspects than the other interviewees, and usually also provide more detailed descriptions.

**Fig. 2:**
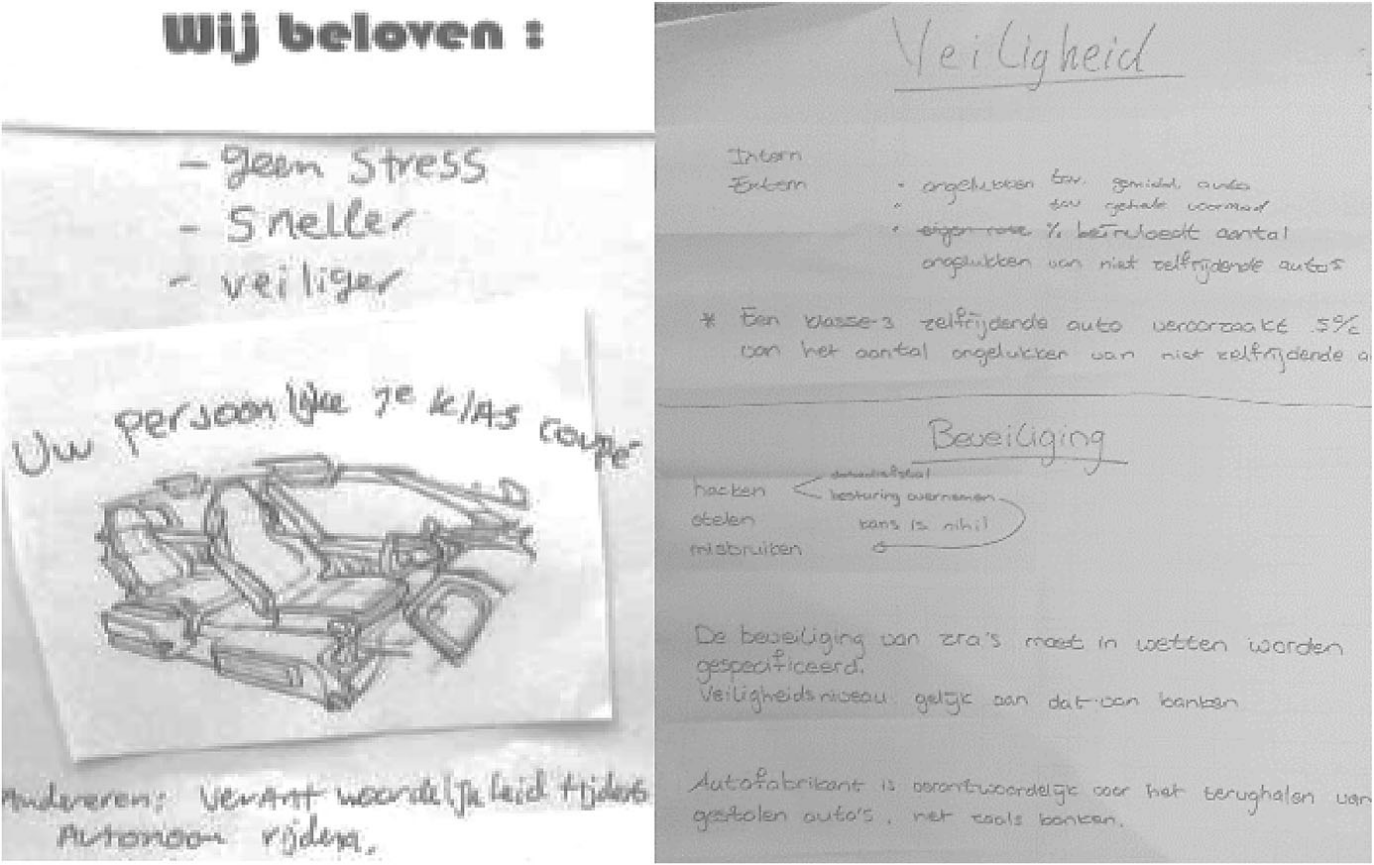
*Left* example of developed flyer for step 1a, in Dutch, that reads (from top to bottom): “We promise: no stress; more quickly; safer; your personal first class coupé; we guarantee that we [manufacturers] are responsible during autonomous driving.” *Right* The A0 with specification details for step 2b. On top safety is specified (‘a level 3 self driving car causes 5% of the number of accidents relative to the amount of accidents of normal cars’), security on the bottom (the security of AVs should be specified in laws and regulations, with a security level similar to that of banks. Car manufacturers are responsible for returning stolen vehicles, similar to banks’)

The creative element worked well with our representatives, but also some of them hesitated when asked to start designing. The A4 paper with starting points was often used; an example of a developed flyer is given in Fig. 2.

Overlapping aspects between interviewees included increased safety, fewer traffic jams, and doing something else while driving. Interestingly, also some value-based dilemmas and differences in viewpoints between actors emerged. E.g., although everyone names an improved traffic flow as important, the manufacturer explains that manufacturers cannot be held responsible for improving the traffic flow. We also observed some empathy between groups. E.g., the non-consumer indicates that he wants development of automated vehicles to be stimulated, although he would not want one himself at this moment.

### 1b. Clustering

In the session with three experts, the 72 statements were clustered into 7 initial value clusters. The experts were first asked to cluster the statements individually into groups, and thereafter the experts and the researchers combined their insights into one set of value clusters. To do this, each expert was provided with a stack of 72 cards, with all the different statements.

Several statements from the interviews were not taken up in the developed clusters. In total 9 statements were left out: three of them were collectively considered not specific enough, two were on the process of the innovation development and not about the form of automated vehicles, and the other four were not clear enough, or not on automated vehicles.

To check if the identified clusters are collectively exhaustive, the clusters were compared to the literature. In several studies ‘aspects’ or ‘features’ of automated vehicles are mentioned, but these are hard to relate to in our study, as they mostly regard level 5 vehicles, and the aspects these papers describe frequently have no empirical or theoretical foundations. Still, all our clusters can be found in literature (see Table 2). However, an eighth cluster was mentioned frequently in the literature, but did not come back in the earlier interviews with stakeholder representatives or expert session: security (Schoettle and Sivak 2014; Kyriakidis et al. 2015; Tech Times 2015; Wilmink et al. 2014).

**Table 2:** The 8 values regarding automated vehicles identified in this research

Value	Description	Literature
Spending time differently	The driver can do something else while driving.	A
Safety	Fewer and fewer serious accidents	A, B, C
Higher traffic flow	Faster and with less emission from A to B	A, B, C
Liability	There is clear regulation and the driver is not liable when the system is on.	A, B, C
Accessible for everyone	Every group in society (also novice drivers or elderly) can understand and drive automated vehicles.	C
Self-determination	The driver, and no other party, can decide on the speed, route and data use.	B
Equality	All road users should benefit from the introduction of automated vehicles, or their position should remain the same.	B
Security	Automated vehicles are harder to be hacked, misused or stolen (both data and the vehicle).	A, C

Found in the literature: A: Fraedrich and Lenz (2014) clustered reactions under news websites in Germany (from Bild to the Süddeutsche Zeitung). B: Howard and Dai (2014) studied 107 likely adopters in Berkley. C: KPMG (2012, 2013) made a report on automated vehicles and clustered the possible advantages

### 1c. Scoring

In total, our questionnaire setup had 144 responses: 119 online, and 25 offline. The response-rate is impossible to determine as links were distributed in mailing lists and via social media. The first respondent filled in the questionnaire on 17th of September 2015 and the last the 19th of November 2015. The respondents were not evenly distributed over the actor groups, with 7 manufactures, 9 government representatives, 58 possible consumers, and 70 possible non-consumer, i.e. other road users (but again, we do not wish to claim validity and reliability, just the approach). 78 of the participants were in the age category 21–30, 6 were younger, and 35 were older, and 25 unknown (i.e. the offline participants).

**Fig. 3:**
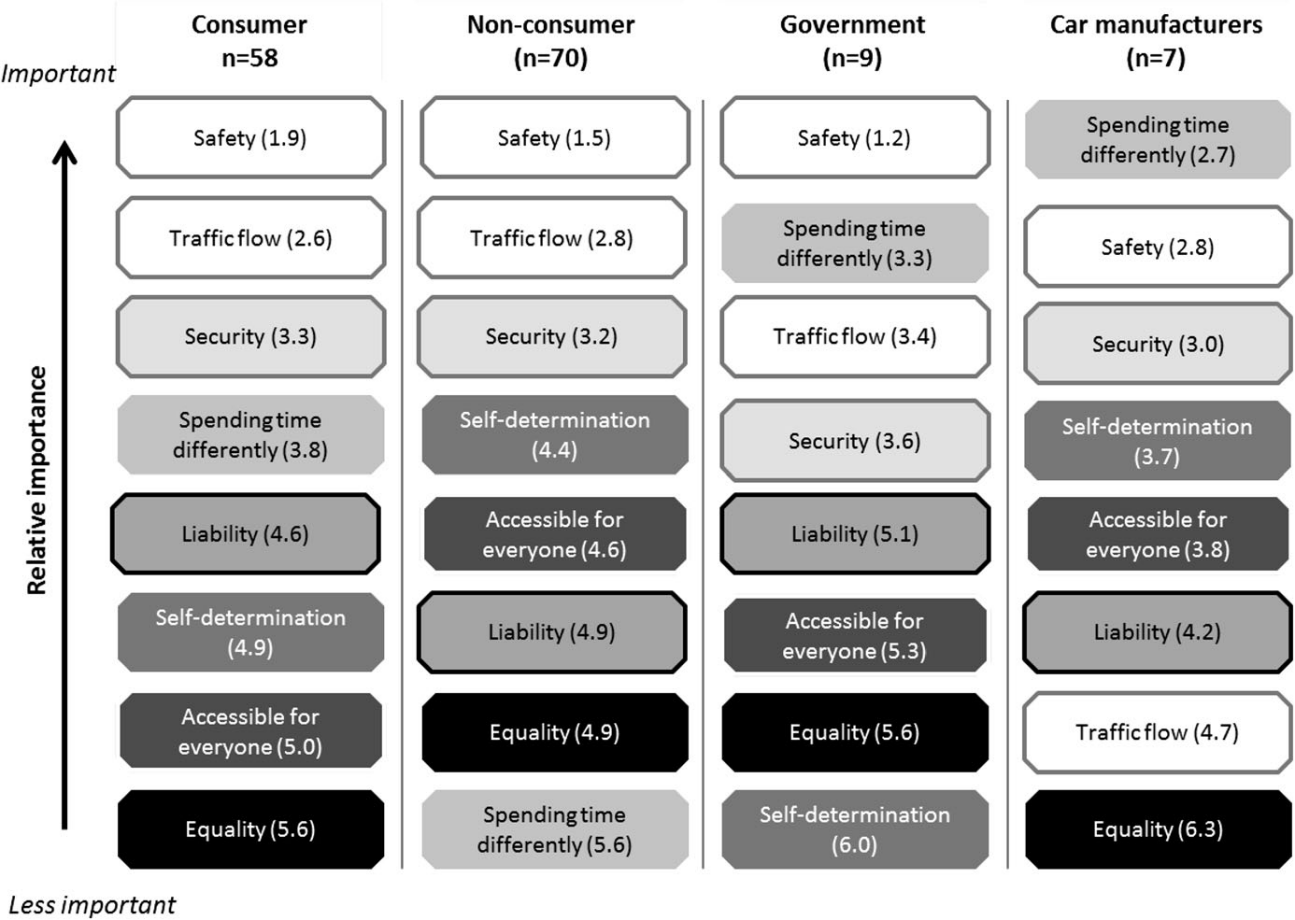
Relative importance of the values per actor. The same values have the same colours. The number behind the value name is the average rank for the stakeholder group, i.e. the value of 1.9 indicates that on average the value is at rank 1.9

As we explained in the method section, the sum of the points is not scientifically correct to use, as the data is ordinal. Therefore, the rankings (i.e. ordinal data, to draw conclusion upon) are shown. To get an idea about the score distribution, also the average scores per group were calculated. In Fig. 3 We show ranks of the scores by different actors (on top of one another), as well as the average position of these values in the group (the value in brackets).

The value profiles of the different actors do not differ much, mostly no more than two ranks of difference. However, there are two striking differences between the actors. First, safety is indicated as the most important aspect, except for the car manufacturers who score this value lower than the other groups. Since car manufacturers have most influence on safety, this is a point to highlight (still, as we will show below, the standard deviation for manufacturers on safety is very high, which means that not all manufacturers may feel the same). Second, a similar pattern can be seen in traffic flow. Three actors score this value high, but car manufacturers connect less value to this point.

To check if a group has an unambiguous opinion, the standard deviations of the rankings within the groups were calculated. Table 3 shows that our standard deviations are quite high. Several reasons could explain these large standard deviations, apart from the small sample size for two of our groups of respondents. First, the values might be misinterpreted. But, easier values to grasp, like *spending time different, safety* or *security* and possibly more difficult values like *self*-*determination* or *equality* do not underpin this last point, as they all have high standard deviations. Second, people may answer the questionnaire while having different interpretations of the values. To test if people may have different interpretations of certain values, we checked their answers to the questions as to why they ranked certain values high, and others low. Only for the value *self*-*determination* and *traffic flow* we found indications of this. Regarding the value *self*-*determination*, we found that respondents felt that this has many aspects, some of which might contradict. Privacy, data ownership, fun of driving and distrust of the automating system are all mentioned together in this value by the respondents. Also, in one of the offline questionnaires a respondent indicated that perhaps traffic flow would have gotten more points if sustainability was more clearly articulated in the questionnaire. So, there might be a slight misalignment for these particular values.

**Table 3:** Standard deviations for the ranks per aspect per actor

Value	Consumer	Other road user	Government	Car manufacturer	Whole sample
Spending time different	2.3	2.0	1.9	1.9	2.3
Safety	1.2	0.9	0.4	2.2	1.0
Security	1.6	1.8	1.9	2.0	1.8
Traffic flow	1.9	1.7	1.8	1.8	1.8
Liability	1.8	2.0	1.9	1.5	1.9
Accessible for everyone	1.9	1.8	1.4	1.7	1.8
Self-determination	1.9	1.9	1.3	2.8	2.0
Equality	1.7	2.1	1.7	1.2	1.9

But most likely, third and last, the opinions within a group may simply differ. Assuming this may be the case, the data shows that most disagreement is on *spending time differently*. Interestingly, fewer internal inconsistencies are found on the value of safety for most actors apart from the manufacturers; they seem to disagree more on that value than the other three actors. For manufacturers their apparent internal disagreement this could be explained by the fact that we asked respondents of different car brands, possibly each with different visions. For the government (where the standard deviations on the ranks are the lowest, but still 1,5 rank) this could indicate that different institution involved find different aspects important. For the consumers and non-consumers this indicates that there is normative diversity among these groups.

### 2a. Scoring Together

In the test session and the two following sessions a common value profile was developed by the workshop participants. During the test workshop and the first workshop the participants created a common value profile, on which they all agreed. In the second workshop it took somewhat longer to create a common value profile. Here more discussions took place on the interpretation of different values, leading to more attention being given to the specification. In any case, the actors did not seem to have opposite opinions, just differences in approaches and priorities.

**Fig. 4:**
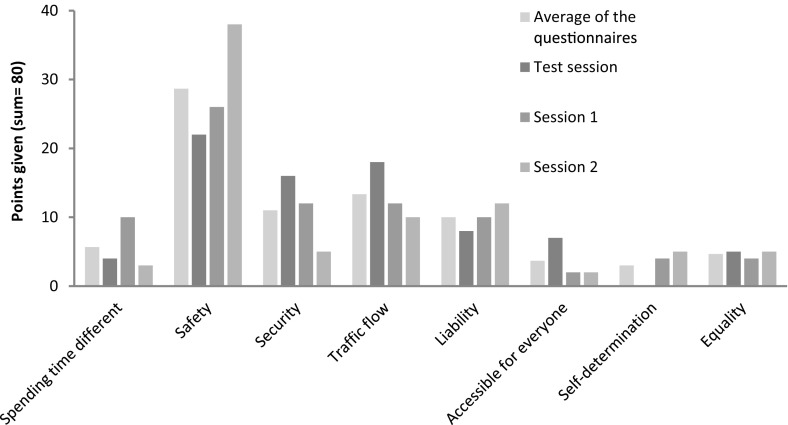
Overview of average scores in the questionnaires vs. in the three organized workshop sessions with engineering students. The ‘average of the questionnaires’ is the unweighted average of all respondents together

The differences in group profiles from the earlier online/offline survey might indicate that most discussion between and within actor groups would be on *spending time differently, safety, traffic flow* and *self*-*determination*. But in the workshops a slightly different pattern could be seen (Fig. 4). The analysed transcripts showed that most discussion was on *spending time differently*, *traffic flow* and *self*-*determination.* Especially *safety* (which had a high standard deviation in the questionnaire) and *security* are the topics which the workshop participants immediately agree upon. Also the specification of *safety* or *security* had less discussion than the other values.

### 2b. Specification to Norms

The workshops revealed that they are ways to make the tensions clearer. Not only the tensions between the values as such (‘I find this important’), but also the tension in interpretation within one value (‘for me this aspect means’) become clear. These tensions during the workshop led to multiple discussions. Most discussions were about *spending time differently, traffic flow* and *self*-*determination*. In most cases there was a struggle between the manufacturer and user on one side and the non-user and government on the other.

Both the *value judgements* and the *disagreements* on them became clear from the workshops. Different groups judged aspects differently. In the second session, the non-consumer raised the point that he would like to add the ‘the feeling of safety’ to the value of *safety*. In the case of *self*-*determination* some parties value privacy, whereas others value the autonomy of the driver. Apparently through discussion, further specifications of the value into the directions of norms became apparent.

Three examples of such specification include, first that the value *safety* was translated to the norm of *fewer accidents.* This then became a design specification: *automated vehicles will cause* 95% *fewer accidents than current cars.* A second example of a specification is that the value *traffic flow* was translated to the norm of *less congestion.* This then became a design specification: *at least the same travel times for non*-*users as in the current situation.* Third, the first group came with original ideas to relate the technology to, e.g., *security* was specified to “*on the same level as a bank”* (also see Fig. 2). In the second session more traditional relations were used, but the outcomes were more quantified and more aspects were discussed.

### Constructive Dialogue Elements

In Sect. 2 we highlighted that for constructive dialogue, there should be elements of ‘constructive’, ‘dialogue’, and other innovation specific aspects. Our closed coding of the discourse on the aspects identified earlier, showed that topics are set by the participants, questions are asked and there is much discussion on the values, their interpretation, and how they could and should be transformed into design requirements. All participants were free to contribute, and did this. Our codes show indications for equality in the discussion: a relatively equal contribution of all participants. The aspect ‘mutual trust and respect’ is hard to derive from the codes, as showing respect is hard to analyse from transcripts. Therefore, the opposite was investigated: being disrespectful. This type of behaviour was not found in the transcript codings. The aspect ‘openness’ was observed frequently, especially in the first part of the workshop: participants are frequently open about their values and standpoints, and even help each other in formulating arguments or help each other with coming up with arguments. Also the participants display a constructive and cooperative mind-set. One example is the non-consumer who explained in the first session: *“Let’s search common ground. First more points there [on safety] and there might be points which no one scored yet, but we now want to score together”.* The argumentative quality is generally high, but we did find some examples of people using powerful normative statements. E.g, a consumer stated: *“If I pay for the car, I want to get something for my money. Otherwise I’ll stay in my current car”*. Regarding the reflective nature of the dialogue, this is hard to assess, as the workshops were done with students.

Also, the workshop can be called ‘constructive’, i.e. aimed at influencing technological choice and design processes. Our method ensured that the values become practically tangible through design requirements, and even new ideas arise in the workshop.

Regarding the innovation specific criteria of constructive dialogue, the use of values and norms still gives *freedom for the designers* to give their own interpretation. Manufacturers still need to formalize and fuse the specified norms and design requirements into actual designs. Of course, there is still the freedom to do nothing, but possibly the form and specificity of the results provide more incentive for action than earlier examples of critical reflections with potential users only based on values, without further specification.

The method also overcomes the problem that there is no clear single collective image of self-driving cars by the participants. The method provides input instead of solely reflecting and criticism, which is probably needed in this phase of the development. The approach also allows for a common language to be found between experts and non-scientific experts through the shared value descriptions and development of design specifications.

## Discussion & conclusion

This paper aimed to present a method that helps innovators to identify important societal values, and involve external stakeholders in their innovation process to come to a co-creation process for the design requirements of new technology. We do so in a format in which the identified viewpoints of the actors can be valued, and even appreciated through active inclusion in design practices. In our two-step method, we first develop value profiles of a group of actors that should be involved in such a co-creative process, and second, organize a workshop in which the actors can collaboratively discuss these values and transform them into norms and design requirements.

The basis of the method is a combination of the value specification processes in Value Sensitive Design, the communication structuring of Midstream Modulation, and the combined use of contributory and interactional expertise, involving non-scientific experts in a design workshop. While VSD methods in literature are often used and meant for one expert group (e.g. a developer) and one laymen group (e.g. the potential consumer), we involved two expert groups and two non-technical expert groups. Of course, VSD has mainly been used in the field of software development, and MM has mainly been used in nanotechnology and biotechnology. Still, in our observations we did not find any indications as to why our VSD and MM inspired approach would *not* work in the area of automated vehicles. The innovation is concrete enough for participants to contribute to design requirements in a constructive way, and the workshop format and supervision appears to provide ample opportunities for people to participate.

Still there are some remarks that should be made with regard to our approach. Whereas in VSD research the values and their interpretations stay qualitative, here they are used quantitatively using a point-division system. Of course, making values quantitative may not completely respect the nature of values, but it gives a good indication, and, as we show, a valuable starting point for discussions with engineers. By allowing many people from the four different groups to fill in the initial questionnaire, the differences between the groups were made more explicit.

The difference between the questionnaire results and the value profiles developed in the workshop can be explained by the fact that during the workshop, arguments can be articulated that allow for more nuanced viewpoints. However, we did not observe completely opposite viewpoints in our study. It could be problematic for our approach if two actors have completely opposite opinions, e.g. because of hostile tensions between stakeholder groups. We feel that an initial survey would bring this to light, and if observed, then possibly our approach is not the most appropriate one to follow.

Due to the distribution method of our questionnaire, the sample probably contains a bias to higher educated people with a technical background (as members of the newsletters used for survey invitations, as well as the used social media channels). As a result of these biases and the relative small number of people having filled in the questionnaire, the survey is not fully representative. However, that was also not our aim, and it still gives it gives a good indication of how the method worksThe government representatives and car manufacturers seemed to be the most difficult to reach; most of these contributed in the offline form of our survey method. As such, this seems to be a valuable way to collect data from these stakeholder groups.

The results of the survey demonstrated that there are differences between the groups, but also within the groups the differences are high. The high standard deviation within the groups was not initially expected, but can be explained by the fact that even though we attempted to create shared understanding with an initial instruction video, people within one group still have different interpretations of the identified values or just have different opinions on these values and their importance. Also we asked different members of different governmental organizations and car manufacturers. These organizations may simply have different ideas about the qualifications and design requirements of automated vehicles. It might be interesting to research if different groups within the groups can be found. Possibly, follow-up research could consider using Q-methodology (Brown 1996) to differentiate such groups and create personas.

We also want to stress that the creative interview session using design criteria, proved to be valuable in this type of value research. It shows potential for VSD research in other fields of technology. Probably the method works less well for value research for currently existing products or situations, as imagination is of less importance. Also, we wonder how this approach would work for innovations that are less concrete (smart grids, or nanomaterials) with a less direct and large influence on societal context, such as automated vehicles and the infrastructure around them. We would of course welcome new research focused on such innovation contexts.

Still, the embedding of the method in a business or policy making setting has to be investigated and the effect on actual engineering design should be confirmed. At this moment the method seems to work, but it can only have a real effect in policy or engineering practice if the outcomes are actually used. As we could not gather real experts for the final part of our workshop setting, investigating its real time effects was out of the scope for this current research, but our method should be easy enough to apply in real settings for those who wish to use constructive dialogue workshops. Something interesting to discuss when instead of students, real actors would participate in our constructive dialogue workshop, is the question what the participants other than the car manufacturers get in return. After any constructive dialogue event, the ‘other actors’ (i.e. not the innovators) should not merely be thanked for their contribution and be sent on their way. Some concrete follow-up in which innovators demonstrate the value of these contributions in practice, is probably advisable. Theoretically, such a situation might have aspects in common with trading zones (Galison 1997), and the challenge will be to set up a situation that enables necessary critical interaction and exchange in a way that still has characteristics of ‘constructive’ and ‘dialogue’ as described above. What kind of ‘trading’ is necessary, and what kind of value is actually exchanged, remains to be discussed but it probably differs in various contexts. However, this is not the focus of this current paper, but would still be interesting to study as a follow-up of this project.

Lastly, our approach surpasses the idea that automated vehicles are a heuristic innovation to work on, to begin with. One could argue that value discussions geared towards design requirements move beyond the idea that automated driving is the right future for the transportation sector. We would like to make clear that such value discussions about the basic principles of transportation innovation are indeed valuable, and that innovators, government representatives, possible users and non-users can probably have a fruitful discussion on such premises. We stress that our type of value discussions are of a particular type, possibly more valuable for designers of automated vehicle technology. Still, we invite others to verify if our proposed approach can also be used to discuss the premises of social desirability of innovation.

## Footnotes

1 For our example: https://www.youtube.com/watch?v=xnTHUstmvs4.

2 Of course, it would be preferable to have real actors involved, and we encourage others who are trying this approach to organize that. However, for the purpose of this research project, unfortunately we didn’t manage to get real experts around the table. To increase the reality of the sessions, students with a relevant background in automated vehicles were found who were willing to join.
